# Presenilins and the γ-secretase: still a complex problem

**DOI:** 10.1186/1756-6606-3-7

**Published:** 2010-02-05

**Authors:** David H Small, David W Klaver, Lisa Foa

**Affiliations:** 1 Menzies Research Institute, University of Tasmania, Hobart, Tasmania 7001, Australia; 2 Dept. Biochemistry and Molecular Biology, Monash University, Victoria 3800, Australia

## Abstract

The presenilins form part of a complex of membrane proteins that are involved in the proteolytic cleavage of cell-surface molecules. This article reviews the history of the discovery of the presenilins, their role in the pathogenesis of Alzheimer's disease and in the metabolism of the amyloid-β precursor protein. Unanswered questions about their biochemical mechanism of action and their effects on Ca^2+ ^homeostasis are examined.

## 

Alzheimer's disease (AD) is the most common cause of dementia in the elderly. Typically 5-10% of the population over the age of 65 have dementia, and of these cases, a large percentage have AD [1]. AD is characterised by the presence of proteinaceous deposits in the brain [2]. The extracellular amyloid deposits, which are found in the neuropil (amyloid plaques) and in association with small-medium size cerebral blood vessels (cerebral amyloid angiopathy), are composed of a 4 kDa polypeptide known as the amyloid-β protein (Aβ) which is derived by proteolytic cleavage from a much larger amyloid-β precursor protein (APP) [3]. Aβ displays a spontaneous ability to aggregate into oligomers and larger fibrillar structures, and it is generally thought that the accumulation of oligomeric Aβ is chiefly responsible for the neurodegeneration that occurs in AD [4].

For the generation of Aβ, APP is first cleaved on the N-terminal side of the Aβ sequence by the β-site APP cleaving enzyme-1 (BACE1), a transmembrane aspartyl protease [3]. The resulting 99-amino acid residue C-terminal fragment (C99) is then cleaved by the γ-secretase to yield Aβ and a C-terminal APP intracellular domain (AICD) fragment (Fig. 1). The function of the AICD fragment is unclear, although it is thought to have a role in intracellular signalling. For example, AICD may be involved in the regulation of gene transcription, synaptic plasticity and cytoskeletal dynamics [5].

**Figure 1:**
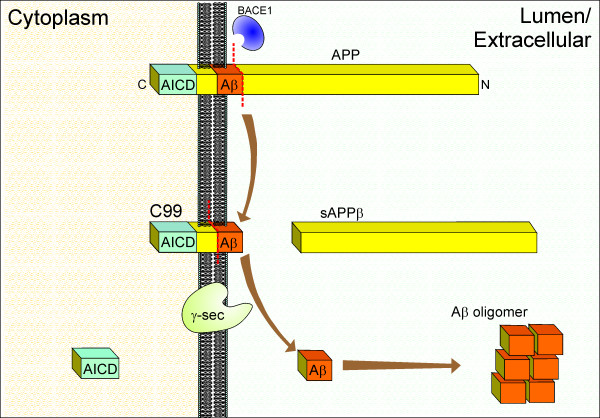
**Amyloidogenic processing of the β-amyloid precursor protein (APP) by BACE1 and γ-secretase**. Initially, BACE1 cleaves APP on the N-terminal end of the Aβ sequence to yield a large secreted N-terminal fragment (sAPPβ) and a smaller membrane-associated C-terminal stub (C99), which is then cleaved by the γ-secretase complex to yield Aβ and an APP intracellular domain (AICD). Secreted Aβ aggregates in the extracellular environment to form neurotoxic oligomers.

The major form of Aβ possesses 40 amino-acid residues (Aβ_1-40_). However, other minor species are also produced which vary in the C-terminal sequence. Production of a longer 42-residue species (Aβ_1-42_) is thought to be intimately associated with AD pathogenesis [6]. Aβ_1-42 _aggregates more readily than Aβ_1-40_, and increased production of Aβ_1-42 _may seed aggregation of Aβ_1-40 _or other Aβ species [4].

## Genetic clues to the pathogenesis of AD

Approximately 5% of all AD cases are autosomal dominant [7]. Soon after the complete APP sequence was cloned in 1987 [8], it became clear that at least one familial AD (FAD) locus was located on chromosome 21 [9] and attention turned to the APP gene that had previously been localised to a region within chromosome 21. The first FAD mutation was identified within the APP gene [10], and soon after, a number of other APP mutations were also identified [11-13]. All of the FAD mutations in the APP gene cluster around the region encoding the Aβ sequence, suggesting that they have some effect on the aggregation or proteolytic processing of APP.

APP mutations on chromosome 21 account for only a small fraction of the total number of FAD cases. It was clear that multiple FAD loci existed on other chromosomes. The first evidence for an FAD locus on chromosome 14 [14] came well before the identification of the locus on chromosome 21. Then, in 1995, FAD mutations in two presenilin (PS) genes located on chromosome 14 (presenilin-1, PS1) and chromosome 1 (presenilin-2, PS2) were reported [15-17]. The PS genes encode proteins that are homologous to the *C. elegans *sel-12 gene, which is known to be involved in Notch signalling [18]. This observation provided that first clue that PS1 may be involved in cell-surface receptor signalling. To date, >100 FAD mutations have been found in PS1 and 11 mutations in PS2 [19]. The PS genes encode proteins with 8 or 9 transmembrane domains. The proteins are synthesized as ~50 kDa proteins that are subsequently cleaved by a presenilinase into a ~30 kDa N-terminal fragment and a ~20 kDa C-terminal fragment, which remain associated with each other [20].

PS mutations are linked to γ-secretase activity [21]. Specifically, it has been observed that FAD mutations in PS1 increase the proportion of C99 that is cleaved by γ-secretase at position 42 of the Aβ sequence. The altered cleavage pattern causes increased production of the more pathogenic Aβ_1-42_. While the first impression may be that these are "gain-of-function" mutations, such a conclusion is difficult to reconcile with the large number of FAD mutations that have been identified, particularly in PS1. Instead, mutations in PS are more likely to be "loss-of-function" mutations [22] in which a decrease in the rate of γ-secretase cleavage of APP leads to an increase in the proportion of Aβ_1-42_. PS1 knockout has been shown to cause an 80% decrease in Aβ production [23], while combined PS1 and PS2 knockout abolishes γ-secretase activity and hence Aβ production [24]. In addition, γ-secretase activity co-purifies with a high molecular weight complex that contains PS1 and several other proteins (nicastrin, aph-1 and pen-2). It is now known that the γ-secretase consists of a complex of proteins of which PS, nicastrin, aph-1 and pen-2 are the principal components. Expression of all 4 proteins in cells is necessary for γ-secretase activity [25].

Inhibitor studies demonstrate that the γ-secretase is a member of the aspartyl protease family [26]. All members of this family require two aspartyl residues for enzyme activity [27]. Some aspartyl proteases (e.g. BACE1) have two aspartyl residues within a single subunit, but other proteases have only one aspartyl residue, and therefore dimerization is needed to activate the enzyme. The amino-acid sequence of both PS1 and PS2 contains two conserved aspartyl residues within two domains predicted to be membrane spanning. These two aspartyl residues are thought to form part of the catalytic domain [28]. In support of this idea, mutation of these two residues has been shown to cause loss of γ-secretase activity [29]. In addition, affinity labelling experiments demonstrate that γ-secretase inhibitors bind directly to PS [30,31].

While the exact number of γ-secretase substrates is unknown, a large number of transmembrane proteins are reportedly cleaved by the enzyme [32,33]. Some of the γ-secretase substrates (other than APP) include APLP2, Notch, Delta, and tumour necrosis factor-α converting enzyme (TACE). Of these proteins, Notch and Delta may be the most important as some abnormalities or toxicities associated with γ-secretase inhibition or knockdown could be due to failure of the Notch/Delta signalling pathway [34]. Cleavage of Notch by γ-secretase produces a Notch intracellular domain fragment (NICD), the counterpart of the AICD produced from APP. Like AICD, the NICD has an important signalling functions. For example, NICD can translocate to the nucleus where it activates the transcription factor CBF1/JBP-Jkappa, regulating downstream gene expression [35]. Because γ-secretase inhibition could lead to unwanted side effects or toxicities, its potential as a therapeutic target for AD is uncertain. Unless a method can be found to inhibit γ-secretase processing without inhibiting other proteolytic cleavage events, it may be difficult to develop a successful AD therapeutic based on γ-secretase inhibition,.

However, there may be ways around this problem. For example, if a successful AD therapy can be achieved by only partially lowering Aβ production, rather than by abolishing Aβ production, then it may possible to use doses of a γ-secretase inhibitor that are low enough to produce sufficient inhibition of the γ-secretase for therapeutic purposes, but which avoid some of the unwanted side effects. Such a strategy could conceivably be employed in combination with other anti-Aβ agents (e.g. β-secretase inhibitors), if they are available.

## Unanswered questions about PS

While there is now clear consensus that PS forms part of the γ-secretase complex, there are still many unanswered questions. One question is how the PS family of proteins evolved. Although there are superficial morphological similarities between PSs and some other proteases, the PSs and their homologues do not share any significant amino-acid sequence homology with known proteases or hydrolases. Presumable, any similarities PS shares with other aspartyl proteases (e.g. mechanism, substrate specificity, inhibitor profile etc.) must have arisen through convergent evolution. The evolutionary history of the PSs is further complicated by the finding that a PS homologue is present in plants where it performs functions apparently unrelated to γ-secretase activity [36]. Is it possible that PS has a function unrelated to proteolytic cleavage? There is certainly evidence for this idea (see below).

A second question is how intramembranous proteolysis of APP occurs. Both the scissile bond in APP and the active site aspartates in PS are buried deep in the lipid membrane [28], and it is difficult to understand how hydrolytic cleavage can occur in such a non-aqueous environment. Attempts to determine a 3-dimensional structure for the γ-secretase complex using cryo-electron microscopy have not yet resolved this issue [37,38].

Four major families of proteins which catalyse regulated intramembranous proteolysis (RIP) have been reported, the presenilin/γ-secretase family and the signal peptide proteases, which are aspartyl proteases, the site 2 proteases (metalloproteases) and the rhomboid proteases (serine proteases) [39]. The crystal structure of a rhomboid protease [40] provides some clues as to how the γ-secretase could cleave APP. It is clear that the active site in the rhomboid proteases is not buried deep within the lipid bilayer, but is instead formed by a V-shaped opening that faces laterally on one site of the lipid membrane and is exposed to the aqueous environment. The structure of rhomboid proteases suggests that substrates may extend into the catalytic domain where they are cleaved. Thus, although the region that is cleaved may be within the transmembrane domain, the scissile bond must be outside a lipid environment for cleavage to occur.

**Figure 2:**
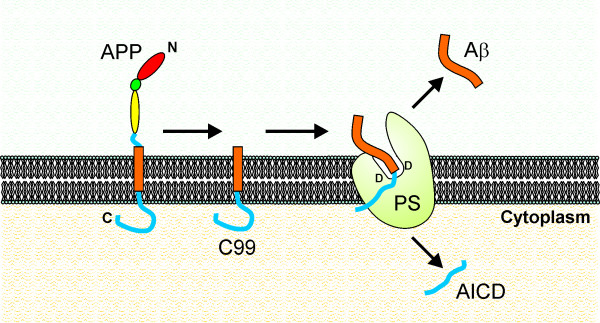
**Hypothetical model showing how γ-secretase/PS may cleave C99 to yield Aβ**. In this model, PS contains 2 catalytic aspartyl residues (D) that form part of the active site. The two residues are in an aqueous environment formed by a pocket on one side of the membrane. After cleavage of APP by BACE1, the product, C99, is destabilised and slips into the active site where it is cleaved to form Aβ and the APP intracellular domain (AICD).

A similar mechanism could occur within the γ-secretase/PS complex (Fig. 2). Indeed, the idea that the transmembrane domain of APP must be partially uncoiled in order to be cleaved is quite attractive, because it could explain why α-secretase or β-secretase cleavage occurs first, before γ-secretase cleavage. It is tempting to speculate that α- or β-secretase cleavage could destabilise the structure of APP within the membrane, causing the protein to slip in such a manner that it can enter the active site of the γ-secretase complex (Fig. 2).

The possibility that C99 needs to partially slip out of the membrane may also explain why γ-secretase cleaves APP at multiple sites. While the commonly held view is that γ-secretase cleavage involves the two main cleavage sites (positions 40 and 42 of the Aβ sequence), the actual cleavage pattern is much more complex. Several different cleavage sites close to the C-terminal end of the Aβ sequence have been identified. For example, several C-terminally truncated Aβ species can be produced, indicating that other cleavage sites exist [41]. It might be expected that if the first γ-secretase cleavage occurs at position 40 or 42, that the AICD fragment would then commence its N-terminus at position 41 or 43. However, this is not the case. Most studies indicate that AICD begins at or close to position 49, which is referred to as the ε cleavage site [42]. In addition to this site, a ζ-cleavage site has been identified at position 46 [43]. It is highly likely that the γ-secretase cleaves at this ζ-site as well, as ζ-cleavage is inhibited by γ-secretase inhibitors [43]. Cleavage of C99 could occur sequentially with the total amount of slippage of the C99 peptide dictating the final cleavage position. Of course, such a possibility must remain speculative until the idea is tested experimentally.

Another question is whether PSs have functions that are entirely unrelated to proteolytic activity. Numerous studies suggest that PS functions to regulate diverse activities such as Wnt signalling, neurogenesis, cell adhesion, synapse formation and apoptosis [44]. It is not the aim of this review to cover this extremely diverse group of studies, as it has been well reviewed previously [44]. The central question is whether these diverse functions can be explained by a γ-secretase activity. In this context, it is worth noting that some functions of PS do appear to be unrelated to γ-secretase. For example, although PS1 interacts with β-catenin, γ-secretase activity is not needed for this interaction as it is not affected by γ-secretase inhibitors [45].

## The plot thickens - the role of calcium

Despite the evidence that PS forms the catalytic subunit of the γ-secretase complex, another putative role of PS has emerged. Recent studies show that mutations in PS disrupt Ca^2+ ^homeostasis; however, the cause of this disruption remains obscure. Dysregulation of intracellular Ca^2+ ^levels is thought to be an important mechanism in AD pathogenesis [46]. Therefore, PS-mediated effects on Ca^2+ ^may be important for understanding the pathogenesis of AD. However, the question is whether PS mutations cause AD by influencing APP metabolism, Ca^2+ ^levels, or both. It has been proposed that PS is an ion channel in the endoplasmic reticulum (ER) and that the FAD mutations increase ion channel permeability [47]. However, other mechanisms seem likely. Studies have shown that PS-regulates the release of Ca^2+ ^via ryanodine or inositol 1,4,5-trisphosphate (IP_3_) channels [48,49], although, once again, the mechanism is unclear. PS mutations alter PIP_2 _metabolism and regulate cation flux through transient receptor potential M7 channels [49], and more recently, Cheung et al. [50] have shown that PS regulates Ca^2+ ^channel gating via a mechanism involving the IP_3 _receptor. PS mutations may also decrease the activity of the sarco ER Ca^2+ ^ATPase (SERCA) pump [51].

However, it is the link between γ-secretase cleavage and intracellular Ca^2+ ^stores which is the most intriguing aspect. Cheung et al. [50] found that PS mutant-induced enhancement of Aβ secretion can be abolished by IP_3 _receptor knockout, indicating that γ-secretase activity is controlled downstream of PS by IP_3_. How this finding relates to the hypothesis that PS is the catalytic subunit of the γ-secretase needs to be explored further.

## Conclusions

While much progress has been made in elucidating the structure and metabolism of PS, its binding partners and the relationship between FAD mutations and Aβ production, many questions remain unanswered. Experiments involving gene knockout, overexpression and mutagenesis, and affinity labelling argue strongly that PS is the catalytic subunit of the γ-secretase. However, the role of other components of the γ-secretase complex (nicastrin, aph1, pen2) remains uncertain. The mechanism by which γ-secretase cleaves within the transmembrane domain also remains unclear. Does PS cleave in the hydrophobic environment of the lipid membrane, or does the substrate of cleavage (C99 or C83) slip out of the lipid bilayer prior to cleavage? The latter mechanism seems like a real possibility as it may explain why α-secretase or β-secretase cleavage of APP is required before γ-secretase cleavage. It is certainly possible that cleavage at the N-terminus of the Aβ sequence may destabilise the C-terminal peptide, allowing for some slippage within the membrane.

Finally, the role of PS in the release of intracellular calcium stores needs to be understood. Of particular significance here are the findings that FAD mutations influence inositol phosphate signalling and that inositol phosphate signalling can, in turn, regulate Aβ production. While it is possible that FAD mutations have multiple effects which converge on Aβ metabolism, until the precise mechanism by which PS mutations influence Aβ is understood, there will continue to be more questions than answers.

## Competing interests

The authors declare that they have no competing interests.

## Authors' contributions

DHS, DWK and LF wrote this article jointly. All authors read and approved the final manuscript.
